# Graph Neural Network for representation learning of lung cancer

**DOI:** 10.1186/s12885-023-11516-8

**Published:** 2023-10-26

**Authors:** Rukhma Aftab, Yan Qiang, Juanjuan Zhao, Zia Urrehman, Zijuan Zhao

**Affiliations:** College of Information and Computer, Taiyuan University of Technology, No. 79 Yingze West Street, Taiyuan, 030024 China

**Keywords:** Multiple instance learning, Graph, Whole slide images, Graph neural networks

## Abstract

The emergence of image-based systems to improve diagnostic pathology precision, involving the intent to label sets or bags of instances, greatly hinges on Multiple Instance Learning for Whole Slide Images(WSIs). Contemporary works have shown excellent performance for a neural network in MIL settings. Here, we examine a graph-based model to facilitate end-to-end learning and sample suitable patches using a tile-based approach. We propose MIL-GNN to employ a graph-based Variational Auto-encoder with a Gaussian mixture model to discover relations between sample patches for the purposes to aggregate patch details into an individual vector representation. Using the classical MIL dataset MUSK and distinguishing two lung cancer sub-types, lung cancer called adenocarcinoma (LUAD) and lung squamous cell carcinoma (LUSC), we exhibit the efficacy of our technique. We achieved a 97.42% accuracy on the MUSK dataset and a 94.3% AUC on the classification of lung cancer sub-types utilizing features.

## Introduction

The era of histopathology boasts voluminous electronic image records, a present-day reality. Within these records lies an overwhelming wealth of information, as exemplified by the notable study [1]. Yet, the path to accessing and harnessing this accrued knowledge for examination, research, and training purposes remains largely uncharted. The dearth of suitable methods to represent Whole Slide Images (WSIs) compounds this challenge, necessitating a deeper exploration of efficient WSI representation techniques. These endeavors become even more critical given the intricacies involved in depicting WSIs, including factors such as sharpness, features, hues, and pathological clarity.

The advent of deep neural networks has revolutionized digital pathology, sparking collaborative efforts between AI specialists and pathologists to innovate diagnostic approaches. As the digital pathology landscape gains wider acceptance, demanding increasingly effective WSI evaluation, new avenues have emerged. Deep learning has ascended to the forefront of visual computing, surpassing conventional visual interpretation techniques. Nevertheless, the sheer volume of pixels within each WSI presents an insurmountable hurdle for deep neural networks. Recent research has delved into the patch-level analysis of WSIs, necessitating manual annotation by experts. However, applying such techniques to large WSI datasets becomes impractical. Additionally, labels often pertain to the entire WSI rather than individual patches, emphasizing the importance of harnessing information from all patches during WSI representation.

In response, Multiple Instance Learning (MIL) emerges as a promising approach for supervised learning in the context of WSIs. MIL-based techniques initially extract neural network feature-embedded data from image tiles. MIL introduces the concept of “bag training,” employing a collection of bag instances, each with a correlated label, thus offering a pathway to WSI representation. The feature embedding subsequently feeds into an aggregation network to generate slide-level information. MIL techniques, applied extensively in WSI analysis, can be categorized into instance-level and embedding-level paradigms. Instance-level approaches emphasize local information, while embedding-level methods focus on global aspects. Previously, SVM-based models like MI-SVM [2] were common in MIL, but recent complex models like Deep MIML [3], mi-Net [4], TransMIL [5], and attention-based methods such as ABMIL [6] and CLAM [7] have gained prominence. Notably, recent research has spotlighted the application of Graph Neural Networks (GNN) in the MIL framework. Graphs prove effective in modeling histopathology data extracted from WSIs, capturing spatial relationships among entities as nodes or sub-graphs. This graph-based approach excels in capturing both marginal and substantial global information from patches, owing to its inherent potential. Several studies in the field of digital pathology have employed GNN-based approaches to address different aspects. GNNs are particularly suited for MIL problems due to their permutation invariant characteristics, where each instance is represented as a node in a graph. Various innovative methods have been developed, such as HIPT [8], a ViT architecture designed for learning from WSI image topology, and H2Graph [9], which constructs heterogeneous networks for training dense layers. The ProtoMIL [10] method, inspired by by-example reasoning and based on graphical representations, represents another innovative approach to MIL.

This research pioneers a novel GMMConv-based Variational Graph Autoencoder model tailored for MIL applications to WSIs, compressing them into compact graphs. The approach meticulously employs maximum magnification settings for WSIs and incorporates patch-level annotations to highlight individual WSI labels. By representing WSIs as dense graphs, the interpretability of the final representation is greatly enhanced. In this framework, each instance is modeled as a node within a network, facilitating the discovery of interconnections between them. The patches are collected and organized into bags using the sliding window tiling method. Subsequently, a graph structure is constructed from the node features of the stacked patches. The interaction between patches is learned through the Gaussian mixture model representation. This innovative methodology unravels the intricate interconnections among regions while efficiently learning the representation of a given WSI.

To illustrate the efficacy of the model, we conducted classification experiments on two common sub-types of lung cancer, adenocarcinoma, and squamous cell carcinoma. Distinguishing between LUAD and LUSC, expert monitoring is essential. In this article, we leveraged MIL-GNN to perform sub-type classification using WSIs from The Cancer Genome Atlas (TCGA), a freely accessible dataset. Our unique approach employed adjacency matrices to capture interactions between various patches, presenting a novel paradigm for WSI learning with GNN. Ultimately, our proposed method yielded impressive results, achieving an F1 Score of 82.24%, precision, and a 0.943 Area under the curve evaluation.

In summary, our article’s key contributions are as follows:

1. A pioneering graph-based MIL-GNN technique for learning WSI representations.
2. Introduction of an intra-node adjacency layer that fosters end-to-end connectivity among learning nodes.
3. The use of MIL-GNN to identify and predict the most significant patches within WSIs, enriching our understanding of these complex images.

## Related work

Histopathology photos from a whole slide can be as large as 100,000 pixels in size. Annotating such large photos by hand is a time-consuming and labor-intensive process. Recent advancements in machine learning, particularly deep learning [11, 12], have significantly contributed to the field of analyzing WSI. These methods have facilitated notable advancements in various areas, such as disease categorization [13], tissue segmentation, mutation prediction, and spatial profiling of immune infiltration [4, 14–17]. The relevant literature on WSIs representation learning, we discuss in detail below:

**Multiple instance learning (MIL):** There are two main approaches to representing WSIs. The first is sub-setting, where a small subset is extracted from a large pathology image. Despite the requirement for professional expertise and accurate subset extraction, most literature employs this method due to its speed and accuracy. The second technique is tiling, which divides images into smaller, manageable tiles and processes them against one another [18]. The tiling approach can be particularly beneficial for MIL (Multiple Instance Learning) approaches that require more automation. In supervised learning, where each training instance has a label, MIL algorithms assign sets of labeled instances instead of individual ones [2, 19]. MIL techniques can also be applied to learning representations of histopathology images. For WSI analysis, MIL techniques are frequently employed. MIL can be categorized into paradigms at the instance and embedding levels. Instance-level approaches primarily focus on local information, while embedding-level approaches concentrate on global information. Before the advent of deep learning, SVM-based models such as MI-SVM [2] were commonly used to address MIL problems. However, several complex models are now employed to manage MIL. Deep MIML [3] involves training something behind the scenes, which is subsequently pooled to produce a bag representation. mi-Net [4] combines projections from individual instances to generate bag-level predictions. TransMIL [5] efficiently handles balanced or unbalanced data while capturing morphological and spatial information. Specifically, attention-based methods like ABMIL [6] and CLAM [7] can recognize the impact of various instances during global aggregation. Due to the inherent ambiguities and challenges associated with self-labeling, MIL techniques have the distinct advantage of leveraging carefully crafted formations and reducing manual annotation efforts.

**Graph based approaches:** Recent attempts to perform WSI-level analysis have yielded promising results in terms of assessing the microenvironment of the entire tissue.Graph-based methods, namely graph convolutional networks, have drawn significant attention in recent years. This is mostly attributed to their capability to effectively capture the entirety of WSIs and analyze patterns within them, enabling accurate predictions of different outcomes of interest. Recent methods have suggested pooling algorithms for learning hierarchical representations for graph embeddings. AttPool [20] is an example of a paper that uses an attention pooling layer to identify discriminative nodes and construct a coarser graph from the resulting attention values. Model learning was simplified by AttPool’s use of the hierarchical structure, and it outperformed state-of-the-art methods on multiple benchmark datasets for graph categorization. Graph Neural Networks (GNNs) have lately emerged as a prominent topic of investigation in several publications, demonstrating their substantial impact. A number of studies have utilized graph-based methods to analyze WSIs in order to investigate different aspects related to survival analysis [21–24], lymph node metastasis prediction [25], mutational prediction [26], cell categorization [27], and retrieval of significant sections [28]. In the field of digital pathology imaging, Ilse et al. (2018) [6] have successfully developed the permutation invariant operator. Graph Neural Networks have been utilized for MIL problems due to their permutation invariant characteristics. Using each instance as a node in a graph, it was demonstrated that GNN could be applied to MIL. Tu et al. (2019) [29] demonstrated the applicability of GNN for MIL by representing each instance as a node in a graph. In order to categorize WSIs expressed in terms of their constituent pixels, the methods based on GNN have been devised [30, 31]. HIPT [8] created a revolutionary ViT architecture to learn from the intrinsic WSI image topology, whereas H2Graph [9] built a heterogeneous network with higher scales of WSI to train a dense layer. The ProtoMIL method, as defined by Rymarczyk et al., (2021) [10], is an innovative approach to MIL that is based on graphical representations and is inspired by the by-example style of reasoning.

Driven by these recent advancements, we use a learning set or MIL strategy to tackle the problem, disregarding the interdependencies within the sets. Our methodology differs from prior research in its utilization of graph mixture model convolution to depict the connections between bags. In this study, we employ a combination of neural architecture and graph network to comprehensively analyze the bag structure. Subsequently, we proceed to train the acquired layout in a sequential manner, starting from the initial stage and progressing towards completion.

## Materials and methods

**Fig. 1:**
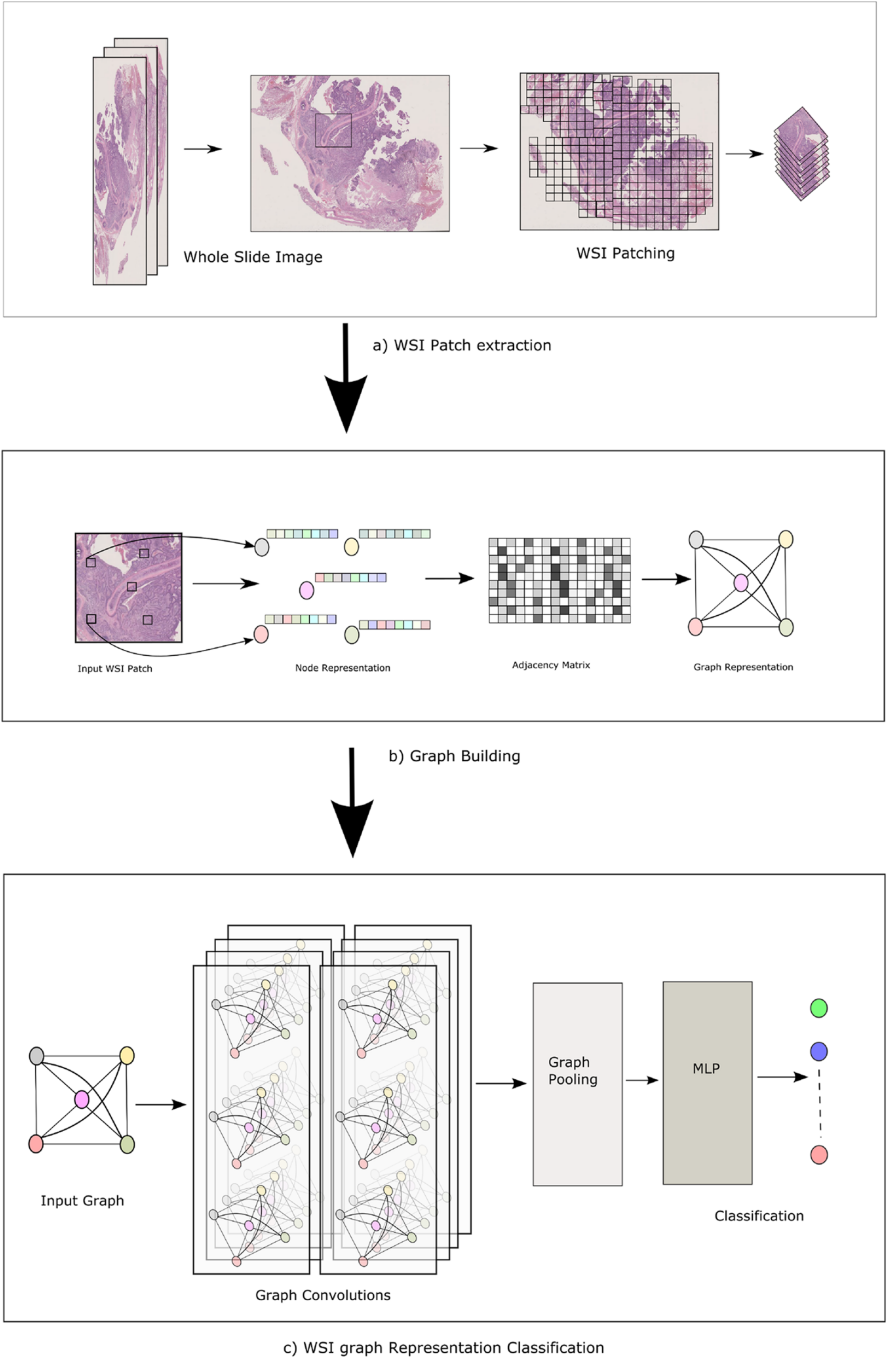
The Overall process of proposed method. 1st row describes building patch using Tiling method. Middle row showing building the graph. And last row showing Deep Graph Neural Network Training procedure

This section presents our proposed framework for acquiring representations of WSI through learning. First of all, we briefly discuss the proposed method based on Variational Graph Auto-encoder (VGAE). The proposed method is memory efficient while training and learning representation that is Non-Euclidean. The proposed approach trains all the way through on a bag of instances to obtain a representation for each patch. The basic concept involves utilizing a graph that is fully connected, denoted by nodes *V* and an adjacency matrix *A*. A graph can represent any model as a variety of relationships. The two standard nodes, denoted as $V_{i}$ and $V_{j}$, are connected by weighted edges represented as $a_{i,j}$. Figure 1 illustrates the overall proposed method. From a WSI, the patches are samples passing through a feature extraction by using a tiling method. All the selected regions’ features are extracted using a convolutional neural network that has already been trained, and those features are then used to build a completely linked graph. The WSI that has been provided is employed as a dense graph. In this graph, each node that is connected is trained to interact with all other connected nodes. After the graph has been pooled, it is sent through a Graph Variational Auto-encoder to produce the WSI’s final representation. The efficient utilization of memory in processing WSI is a fundamental aspect of the process. Final WSI representation has led to classifying applications.

The ongoing research centers on utilizing GNNs to learning graph representations in the context of MIL. The study introduces a new method for addressing the MIL problem. Two phases comprise the suggested approach for expressing a WSI a) Sampling critical patches, arranging them in a fully-connected graph; and b) itemizing the results to classify the fully-connected graph, and permuting it into a vector form. The entire technique could be created in an individual training loop. The significance associated with our technique is the generation of the adjacency matrix, which is a structure that describes the relationships between nodes. The technique can summarize as follows.

1. The present study employs a tiling-based approach to extract noteworthy patches from a WSI. A pre-trained CNN is employed to extract features from individual sampled patches.
2. The provided WSI is thereafter represented as a fully linked graph. The adjacency matrix normally connects every node to every other node. During the Adjacency Layer training block, the adjacency matrix is trained.
3. Subsequently, the graph underwent processing via a GCN, succeeded by a graph pooling layer, resulting in the ultimate vector representation for the given WSI.

**Deep Graph Convolution Layers** We started establishing the VGAE component of the WSI. We tried out two different GCNs, the spectral approach ChebNet and the spatial method GraphMMConv. Each of the GCN’s hidden layers simulates the interaction between the nodes and converts the feature into a new feature space. The next step is a pooling layer, which is responsible for converting the characteristics of each node into a vector representation. Because of this, a WSI may now be represented by a compressed vector, which has the further benefit of being used for a variety of other purposes, like image retrieval, classification, and so on.

**MIL training approach** Our suggested method is applicable across the range in the MIL environment. The steps taken to address MIL concerns are as follows: Each instance is modeled as a vertex, and its characteristics are treated as features. The bag of instances is trained within a global context to calculate features for the adjacency matrix. Each instance as vertex and $a_{ij}$ in adjacency features A that represents the edge weight between $v_i$ and $v_j$ construct a dense graph. The integration of deep graph convolution network facilitates the training of the graph’s representation. This representation is subsequently processed through a graph pooling layer to get a feature vector that represents the bag of instances. The vector of features derived from the graph can be utilized for classification purposes.

**Feature extraction** The study employed a sliding window technique, as described by [32], to generate small patches from the entire slide. These patches were then classified using a residual neural network. The predictions from the patches were pooled, and a heuristic was utilized to determine the predominant and nominal histological patterns for the slide in the whole. Each predicted patch was evaluated separately from its neighbors and from its position in the whole slide.

**Graph building** We suggest a new approach to learning WSI representations of GNN. Each WSI is transformed into a dense graph which has two components node *v* and Adjacency matrix *A*. Each node is representative of a feature vector and correlates with the features extracted from a patch.Conversely, the relationship among nodes *v* is denoted by the Adjacency matrix *A*. Adjacency matrix *A* is learned using patch features in a convolution layer. The training methodology employed in our study involves the iterative learning of the adjacency matrix in a sequential manner, utilizing the *l*2 distance as a threshold for pre-calculated features. We proposed to use context information that connection between two same nodes or patches are uncommon for various WSI. Consequently, the value of an element in the adjacency matrix is contingent upon the links between two patches as well as the contextual characteristics of said patches. We assume *S* be a WSI and $s_1, s_2, \dots , s_n$. The patches went through to feature extraction through a layer, resulting in the derivation of feature representation $x_i$. Then using these features $x_i$ to obtain the context through Zaheer et al [33] theorem. The process of obtaining the context vector involves the utilization of the pooling operator $\phi$ to combine feature vectors from all patches.

1 $$\begin{aligned} c = \phi (x_1, x_2, \dots , x_n) \end{aligned}$$

The context vector c is subsequently subjected to concatenation and MLP layers, resulting in the concatenated feature vector $x_{i}^{\prime }$. This process facilitates the conversion of the new feature vector $x_i^{*}$, which conveys patch information in conjunction with context. To make a form as feature matrix $X^{*}$, features $x_{i}^{*}$ are stacked together. Passing the features through the correlation layer yields the adjacency matrix *A*, where each element $a_{ij}$ indicates the level of correlation between patches $s_i$ and $s_j$. The dense graph representation of WSI employs the notation $a_{ij}$ to denote the weights of edges connecting distinct nodes.

**Deep graph convolution network** We experimented with two types of GCN: ChabNet, which uses a graph neural network, and GMMConv, which uses a Gaussian mixture model convolution operator, to implement the graph representation of the WSI. Within GCN models, every hidden layer establishes connections between nodes and converts the features into a distinct latent space. Ultimately, a layer of pooling is used to combine node characteristics into a solitary vector representation.

**GVAE** We employ a graph convolution network (GCN) [34] encoder and a straightforward inner product decoder. We apply a simple inference model parameterized by two GCN layer

2 $$\begin{aligned} q(Z|(X,A)) = \prod _{i=1}^{N} q(z_i|X,A) \end{aligned}$$

3 $$\begin{aligned} q(z_i|X,A) = \mathcal {N}(z_i|\mu , diag(\sigma _{i}^2)) \end{aligned}$$

where $\mu = GCN_{\mu }(X,A)$ is the matrix of means vectors $\mu _i$. on the other hand, $log \sigma = GCN_{\sigma }(X,A)$. The two layer GCN is represent as $GCN(X,A) = \tilde{A}ReLU (\tilde{A}XW_0)W_1$ with weight matrices $W_i$ GCN mean and average layer share first layer parameters. ReLU utilizes the max operator, and the symmetrical normalized adjacency matrix. Models with inner product between latent variables are generative.

4 $$\begin{aligned} p(A|Z) = \prod _{i=1}^{N} \prod _{j=1}^{N}p(A_{ij}|z_{i}z_{j})\end{aligned}$$

5 $$\begin{aligned} p(A_{ij}=1|z_i,z_j) = \sigma (z_{i}^{T}z_{j}) \end{aligned}$$

where $A_{ij}$ are the elements of A and $\sigma (.)$ is the logistic sigmoid function.

**Loss function** We consider the prior distribution on the random variable *z* to learn the encoder and decoder parameter of VAE i.e., $\phi$ and $\theta$ that models distribution of *x*. So the lower bound can be calculated as

6 $$\begin{aligned} \mathcal {L} = E_{q_{\phi }(Z|X,A)}[logp(A|Z)] - KL[q(Z|(X,A)||p(z))] \end{aligned}$$

where *KL* represents the Kullback-Leibler divergence between *q*(.) and *p*(.), a priori Gaussian $p(Z) = prodip(z_i)$. For sparse *A*, it will be useful to overwrite weight terms with $A_{ij} = 1$ or otherwise zero. For training purposes, we use full-batch gradient descent and the reparameterization method. The identity matrix will be utilized in Graph Convolution Networks (GCN) in lieu of the input feature matrix *X*.

## Experiment

We analyzed the effectiveness of the method using two real datasets MIL public dataset MUSK and TCGA lung cancer slide Dataset. We conducted multiple trials to train and evaluate our model. The TCGA data coupled with the same hyperparameters were utilized as a separate dataset for model testing. On the MUSK1, the proposed method achieved a state-of-the-art accuracy of 93%. Our model was also used to differentiate between two sub-types of lung cancer: Lung Adenocarcinoma (LUAD) and Lung Squamous Cell Carcinoma (LUSC).

**Experiment settings** We initially pre-train a classification network using 10-fold cross-validation, which involves splitting 10 different training sets into 60% training sets, 20% validation sets, and 20% test sets. We next evaluate the network using the area under the receiver operating curve (AUC/AUROC) and weighted F1-Score. Using the ResNet pretrained network and a distinct ImageNet-pretrained model for each fold, we were able to extract features from WSIs of the neural network. The construction of a graph involved the computation of spatial adjacency among the patches, followed by the storage of node-level embedding of the attribute matrix subsequent to the extraction of image features. To incorporate graph convolution networks, we used Pytorch Geometric Library [35] trained using Nvidia RTX3090 GPUs. Each WSI was cropped to produce a set of $512 \times 512$ non-overlapping patches at$20\times$ magnification, with patches from the background whose non-tissue areas were larger than 50 discarded. The CNN backbone used for the feature extractor is Resnet18. We use a mini-batch size of 512, the Adam optimizer, and a cosine annealing approach to our learning rate schedule. The trained feature extractor was kept and used to generate graphs. The model layer’s parameters were $L=3$, MLP size = 123, $D=64$, and $k=8$; we used a graph convolution layer (GCN). Eight samples at a time were used to train the model over the course of 150 iterations. Starting at a rate of 103, the learning rate gradually decreased through steps 30 and 100, final learning at a rate of 105. For a node-level classification on the training slides, we used stochastic gradient descent of Cross-Entropy loss and evaluated our GNN’s ability to generalize on the testing data of the WSIs graph using F1-Score for each cross-validation fold. We kept the anticipated embeddings, predictions on the validation and test sets, and the training epoch with the greatest validation F1-score for each fold of cross-validation.

**Evaluation** To evaluate the efficacy of our approach and cutting-edge techniques, the Area Under the Receiver Operating Characteristic Curve (ROC-AUC), accuracy, precision, recall, and F1-score are used. Specifically,

7 $$\begin{aligned} Accuracy = \frac{TP + TN}{TP+ FP +FN + TN} \end{aligned}$$

8 $$\begin{aligned} Precision = \frac{TP}{TP+ FP} \end{aligned}$$

9 $$\begin{aligned} Recall = \frac{TP}{TP+ FN} \end{aligned}$$

10 $$\begin{aligned} F1-score = \frac{TP}{TP+ FN} \end{aligned}$$

The initials TP, FP, TN, and FN stand for true positive, false positive, true negative, and false negative, respectively. When comparing the performance of various methods, ROC-AUC is the most comprehensive of these.

## Results

**MUSK dataset** The MUSK dataset comprises a total of 92 instance bags, with 47 of them being positive and 45 of them being negative. Instances of the bag refer to the precise shape or characteristics of a molecule. In the event that novel molecules possess a musky quality, we shall discern such dissimilarities in the context of bags. A 10-fold cross-validation was conducted using predetermined arbitrary seeds. In Tables 1 and 3, we presented a comparative analysis of our proposed method against various state-of-the-art techniques. The graph was designed using miGraph’s kernel [36], and it represents the items contained within a bag. The MI-Net [4] and Attention MIL [6] models are implemented using Deep Neural Network architecture and utilize either pooling or attention mechanisms to obtain the bag representation.

**Table 1:** Evaluation of MUSK dataset

Model	Accuracy
mi-Graph	0.889
MI-Net	0.887
Attention-MIL	0.892
Attention-MIL with gating	0.900
MIL-GNN	0.932

**LUAD & LUSC** Non-small cell lung cancer (NSCLC) is a prevalent form of lung cancer, with Lung Adenocarcinoma (LUAD) and Lung Squamous Cell Carcinoma (LUSC) being two significant sub-types. Collectively, these two sub-types constitute approximately one-third of all lung malignancies. Building automated systems requires a number of steps, one of which is the automatic categorization of the two primary sub-types of NSCLC. We were successful in obtaining 1026 diagnostic WSIs from the TCGA archive that is stained with hematoxylin and eosin (H &E), which include LUAD and LUSC. We chose relevant patches from across all of the WSIs with the use of a tile-based patch selection system. Applying ImageNet [37] got image components from these patches and each bag is a set of features characterized as LUAD or LUSC. Our method determines the bags as two lung cancer subclass. The significant AUC rate gained for 10-fold distribution was 0.932 and the average AUC score across all fold was 0.91.

**Fig. 2:**
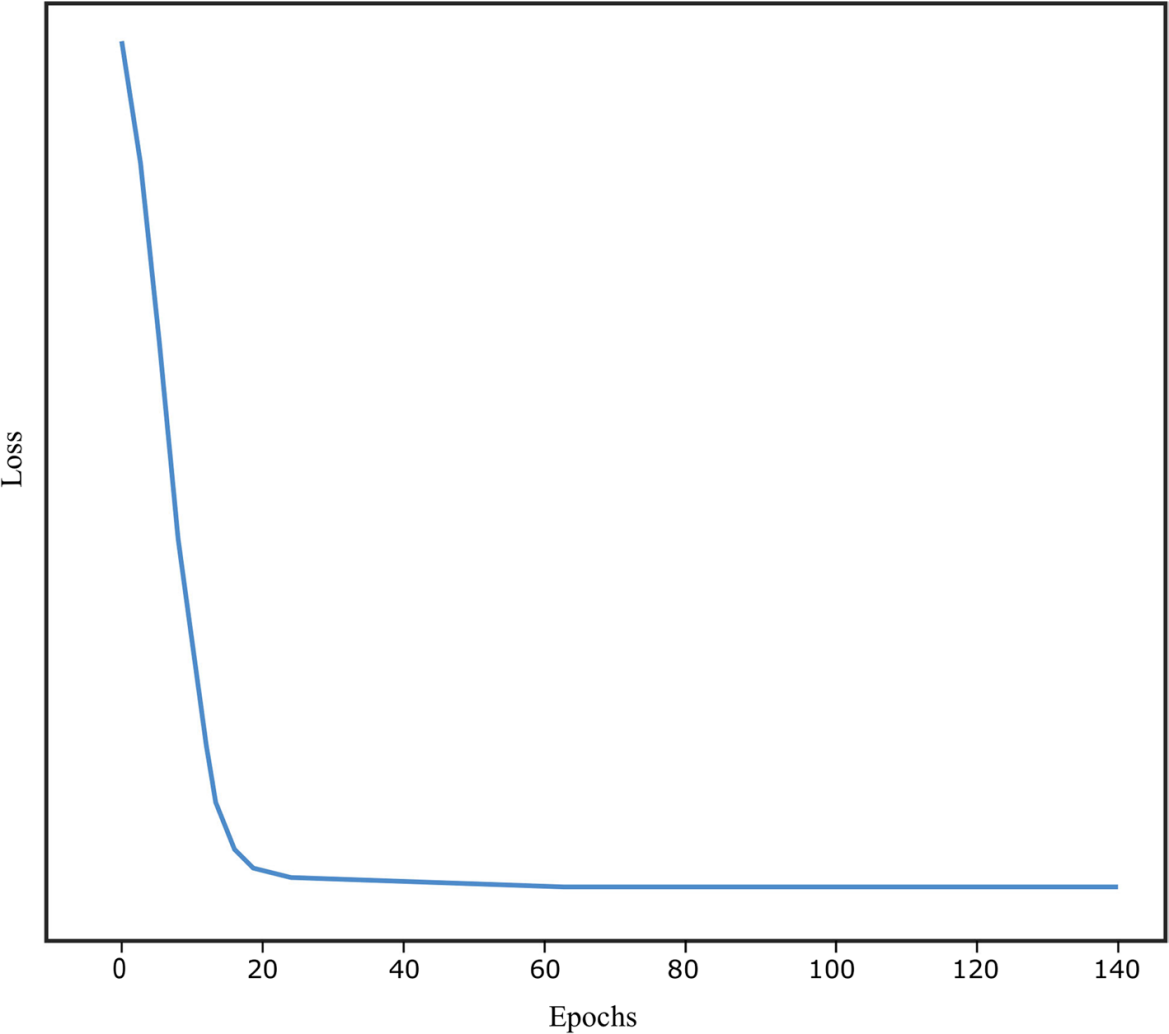
KL divergence loss over epoch

The cross-validation on various subjects is performed, the training performed using WSIs on a distinct class of subjects than the testing. We present the data in tables referred to as Tables 2 and 3. Using a transfer learning approach, we achieved state-of-the-art precision. We squeezed patch components from a prevailing pre-trained network. They will improve the feature extractor during the training. Figure 2 shows the training loss over epochs for each of the folds.

**Table 2:** Evaluation of MUSK dataset

Model	ROC-AUC
Coudray et al. [16]	0.67
Khosravi et al. [38]	0.65
Yu et al. [39]	0.68
MIL-GNN	**0.71**

MIL-GNN has improved 3% ROC-AUC than Yue el at. model on cross- validation data

**Table 3:** Comparison with other methods on MUSK and TCGA dataset

Method	MUSK			TCGA		
	AUC	ACC	F1	AUC	ACC	F1
ABMIL	91.51	86.47	86.33	89.51	82.47	69.75
Gated-ABMIL	93.01	85.03	84.15	89.01	83.03	66.38
MI-Graph	89.7	88.9	78.6	89.7	83.9	70.66
CLAM-MIL	77.10	73.45	73.55	77.10	73.45	72.86
CLAM-SB	92.17	88.66	87.53	92.17	82.66	72.89
GT-MIL	95.92	89.87	89.93	92.92	85.87	77.01
MI-Net	89.03	88.7	86.4	89.03	84.7	75.21
DS-MIL	87.30	81.63	81.05	87.30	80.63	74.25
Trans-MIL	94.24	86.59	86.48	91.24	83.59	76.52
GCN	91.03	89.12	86.14	91.03	84.7	78.4
MIL-GNN	**97.42**	**92.41**	**91.26**	**94.3**	**86.4**	**82.24**

MIL-GNN has improved 3% ROC-AUC than Yue el at. model on cross- validation data

**Fig. 3:**
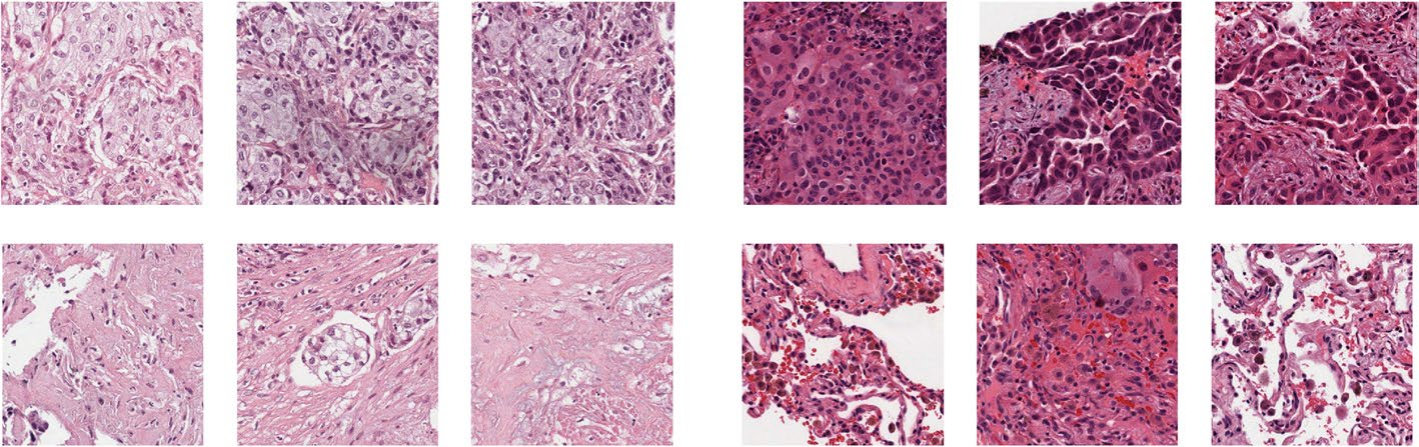
WSI patches with LUSC and LUAD, respectively. Patches are placed such that the top three on the left side are the most significant and the bottom row is less critical, and the opposite is true for the right side

Black-box characteristics of deep neural networks are among the barriers to the functional deployment of advanced neural network models in computational diagnostics. Because our proposed technique uses VGAE, we can observe the weight that our network’s prediction algorithm assigns on each patch. This depiction can provide the pathologist with greater insight into the model’s internal decision-making process. Figure 3 visualize important high-score patches. The global attention pooling layer is taught to award patches an attention score. Higher attention levels imply that the model gives these patches greater consideration. As instances presented for insightful analysis are queued, the CAD system might identify regions of interest and select cases based on analytic requirements. We discover that parts with a higher score for attention contain more nuclei. Because of the fact that morphological characteristics of nuclei are essential for diagnostic decisions [40], the network learns this property.

**Comparison with state-of-the-art methods** We present the results in Table 3, which includes comparisons with ABMIL (2018), Gated-ABMIL (2018), MI-Graph (2019), CLAM-MIL, Deep-Attention MIL (2020), GT-MIL (2021), Trans-MIL (2022), graph message passing (GCN), and our MIL-GNN framework. Across all evaluation metrics, our approach consistently outperforms the others on both the MUSK1 and TCGA datasets. Notably, GT-MIL (2021) stands out as the most effective approach, highlighting the significant impact of the Graph-Transformer architecture in WSI analysis. It’s worth noting that GT-MIL, unlike our techniques, employed Min-Cut pooling and formed a Graph-Transformer network in the task setting.

**Fig. 4:**
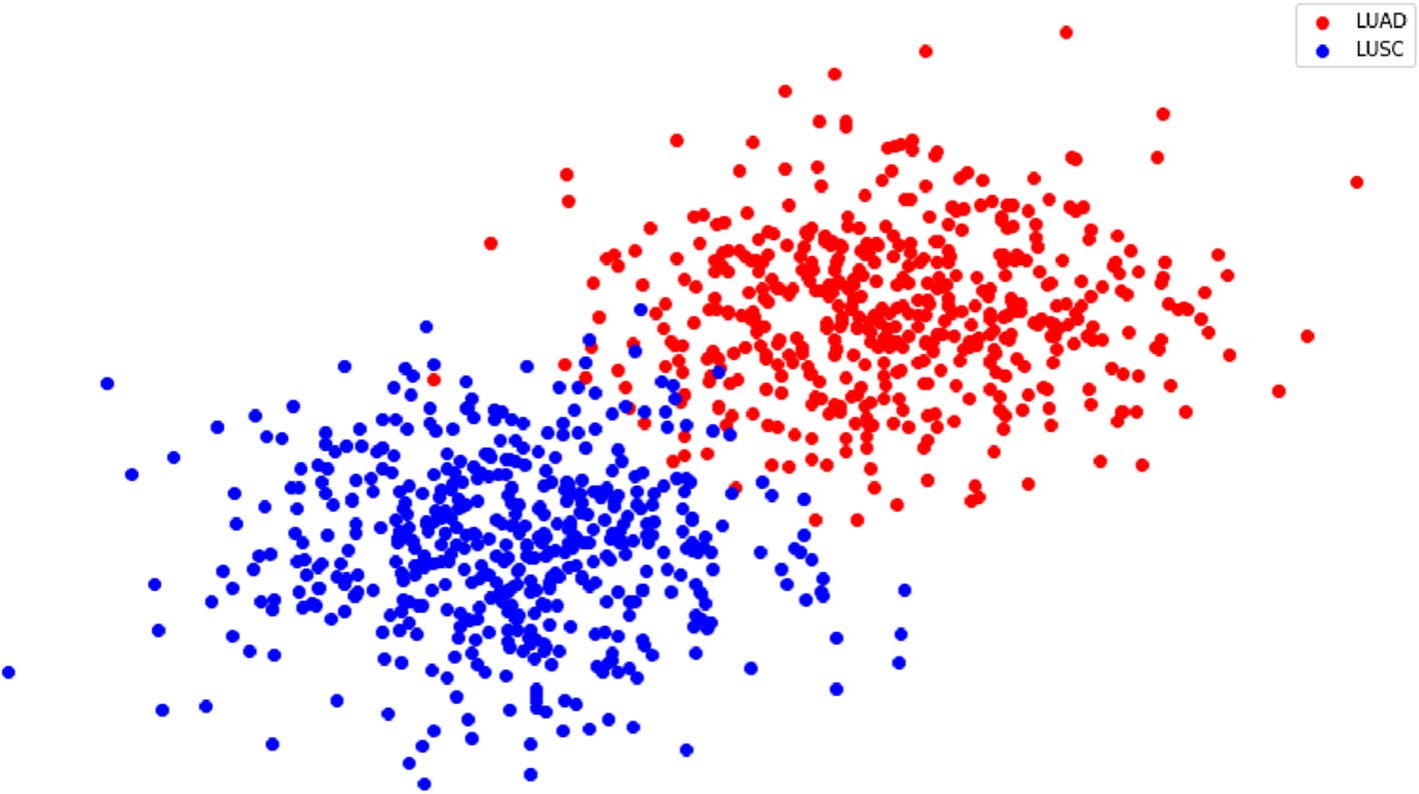
The t-SNE plot for LUAD and LUSC characterized in WSIs

Figure 4 depicts the feature vector of the WSI of the t-SNE plot. It visualized distinct differences among cancer sub-type and showed the strength of our proposed method.

Our framework exhibits notable performance enhancements over GT-MIL, showcasing improvements of 1.50% in AUC, 2.54% in accuracy (ACC), and 1.33% in F1-score when applied to both the MUSK dataset and the TGCA lung cancer dataset. This reaffirms the validity of the models within our framework. However, it’s particularly noteworthy that our framework has significantly improved the classification of cancer subtypes. This achievement is of greater significance, possibly attributed to the robust and adaptable representations learned within the MIL-based graph variational auto-encoder learning paradigm.

**Ablation study** We evaluated the effectiveness of our proposed approach on various configurations of the TCGA dataset. For our Deep Graph Convolution Network, we examined the following layers and configurations: A) MIL-GNN, B) VGAE + GCE, C) VGAE + without GCE, D) with Graph, and E) without Graph. The experiments demonstrated a significant performance advantage of GMMConv over ChebNet. Additionally, we analyzed different encoder layer settings. The results, along with parameter settings, are presented in Table 4. We compared the results obtained when applying the method with and without the creation of a graph.

**Table 4:** Ablation study results

Model Input	Accuracy	Precision	Recall	F1-Score	ROC-AUC
MIL-GNN	0.9274	0.6601	0.8631	0.786	90.6
VGAE +GCE	0.6965	0.575	0.7931	0.666	71
VGAE + without GCE	0.587	0.4902	0.8621	0.6250	67.3
Graph	0.6761	0.595	0.8621	0.5945	68.6
Without Graph	0.4225	0.4902	0.7631	0.5974	60.1

From ROC-AUC accuracy rate, MIL-GNN demonstrate significant improvement with 90.6% than VGAE-GNN. The assessment shows the improvement over the graph feeding. ROC-AUC rates improve With Graph 68.6 where without Graph 60.1. Also, it presents fewer false negatives. Furthermore, this demonstrates that the model can be improved. By completing structural information, performance can be improved.

## Discussion

We’ve developed a graph-based MIL-GNN approach that integrates graph structures to construct a systematic classifier for distinguishing WSIs between LUAD and LUSC. Our method excels when compared to recent architectures utilizing diverse state-of-the-art compositions, as measured by various model performance metrics. Our graph neural network technique adeptly identifies regions within WSIs that exhibit strong correlations with the predicted outcomes. These findings represent significant strides in the field of interpretable deep learning, concurrently advancing the realms of machine learning and digital pathology.

Despite the substantial progress, the domain of digital pathology remains a work in progress, primarily due to the sheer volume of high-resolution images. While these models demonstrate a capacity for accurate predictions, they often fall short in capturing temporal connectivity insights effectively. As a result, the attribution of significant image-level features for deploying such methods may yield mixed results. Our GNN approach effectively addresses this challenge by aggregating WSI-level data into a comprehensive graph structure, marking a notable advancement in the field. The development of a graph that emphasizes WSI regions associated with class labels stands out as one of the distinctive contributions of our research.

Nevertheless, our study does have certain limitations. We made an assumption regarding GNNs’ ability to capture patch-level data and their spatial layout. Additionally, we acknowledge the potential for bias in specific cross-validation folds. Since we stratified patches based on whether they exhibited elevated or decreased prevalence, WSIs may exhibit a range of characteristics, rendering their presence unpredictable. The GNN’s performance suffered due to the presence of numerous unknown parameters. To mitigate this, we employed deep learning to generate patch-level feature vectors before embarking on the design and construction phase, a task that proved to be computationally intensive.

## Conclusion

We present MIL-GNN, a deep learning method based on GMMConv and Variational Graph Auto-encoder, consisting of detection and classification stages. Initially, we convert each whole slide into patch features, treating each patch as a bag to construct a graph by learning the adjacency matrix. Next, we employ a GNN-based embedding design to train the graph model of the VGAE (Variational Graph Auto-encoder) Deep Graph Neural Network. Finally, the resulting representation is fed into an MLP-based classifier to estimate the bag level. The outcomes highlight the superior performance of the proposed technique. We can use an adjacency matrix to visualize relevant patches, making the suggested approach straightforward and interpretable. Future work will explore the effects of deep GNN models with multiple layers. Additionally, we will investigate automatic training and the identification of pertinent histopathological architectural characteristics to obtain semantic features, which present intriguing prospects.

## Data Availability

Musk (Version 1) Data Set https://archive.ics.uci.edu/ml/datasets/Musk+(Version+1) The Cancer Genome Atlas (TCGA) TCGA dataset publically available https://portal.gdc.cancer.gov/.
